# The dynamic network of IS*30* transposition pathways

**DOI:** 10.1371/journal.pone.0271414

**Published:** 2022-07-28

**Authors:** Ferenc Olasz, Mónika Szabó, Alexandra Veress, Márton Bibó, János Kiss

**Affiliations:** 1 Department of Microbiology and Applied Biotechnology, Institute of Genetics and Biotechnology, Hungarian University of Agriculture and Life Sciences, Agribiotechnology and Precision Breeding for Food Security National Laboratory, Gödöllő, Hungary; 2 Department of Microbiology and Applied Biotechnology, Institute of Genetics and Biotechnology, Hungarian University of Agriculture and Life Sciences, Gödöllő, Hungary; Institut National de la Recherche Agronomique, FRANCE

## Abstract

The *E*. *coli* element IS*30* has adopted the copy-out-paste-in transposition mechanism that is prevalent in a number of IS-families. As an initial step, IS*30* forms free circular transposition intermediates like IS minicircles or tandem IS-dimers by joining the inverted repeats of a single element or two, sometimes distantly positioned IS copies, respectively. Then, the active IR-IR junction of these intermediates reacts with the target DNA, which generates insertions, deletions, inversions or cointegrates. The element shows dual target specificity as it can insert into hot spot sequences or next to its inverted repeats. In this study the pathways of rearrangements of transposition-derived cointegrate-like structures were examined. The results showed that the probability of further rearrangements in these structures depends on whether the IS elements are flanked by hot spot sequences or take part in an IR-IR junction. The variability of the deriving products increases with the number of simultaneously available IRs and IR-IR joints in the cointegrates or the chromosome. Under certain conditions, the parental structures whose transposition formed the cointegrates are restored and persist among the rearranged products. Based on these findings, a novel dynamic model has been proposed for IS*30*, which possibly fits to other elements that have adopted the same transposition mechanism. The model integrates the known transposition pathways and the downstream rearrangements occurring after the formation of different cointegrate-like structures into a complex network. Important feature of this network is the presence of “feedback loops” and reversible transposition rearrangements that can explain how IS*30* generates variability and preserves the original genetic constitution in the bacterial population, which contributes to the adaptability and evolution of host bacteria.

## Introduction

Transposable elements are present in virtually all organisms and their activity significantly contributes to remodelling the host genome. Insertion sequences (IS) and transposons (Tn)—forming a diverse group of prokaryotic mobile DNA elements—can transfer genes or larger DNA segments into new genetic locations even between different replicons such as plasmids, bacterial chromosome, or viral genomes. The intracellular mobility of IS elements gives rise to insertions, deletions, inversions, or replicon fusions. These activities may have significant effect on the expression of host genes. Insertion into a gene often results in gene inactivation, while insertion near a gene can modify its expression or reactivate silent genes due to the activity of regulatory elements such as promoters or transcription termination signals delivered by the IS [1–3]. The translocated genes may be placed under the control elements of other host genes localized adjacent to the position of insertion. ISs can contribute to horizontal spread of adaptive functions by mobilizing genes between the chromosomes and transferable elements such as bacteriophages, conjugative plasmids and mobile genomic islands [3]. Furthermore, identical IS copies can also be passively involved in streamlining the genomes by providing homologies for recombination [4, 5]. These capabilities make them important factors in reshuffling the genetic material of bacterial genomes and generating variability, which has a central role in adaptation and evolution of prokaryotes [3, 6–13].

The capacity of an IS for generating wide range of genome rearrangements may depend on the adopted transposition mechanism. Although mechanistically there are few ways to catalyse DNA break and re-joining reactions required for transposition and only three types of catalytic nuclease domains have been described in transposases (Tpase), the key enzymes of ISs, the transposition pathways are more diverse. For example, the most prevalent Tpases having the conserved DDE signature uniformly cut a single DNA strand and generate free 3’OH at the IS terminus. However, the variations in the second strand processing manifest in different pathways described by the classic transposition models such as cut-and-paste, Tn*3*-type replicative or copy-out-paste-in models [14].

**Fig 1:**
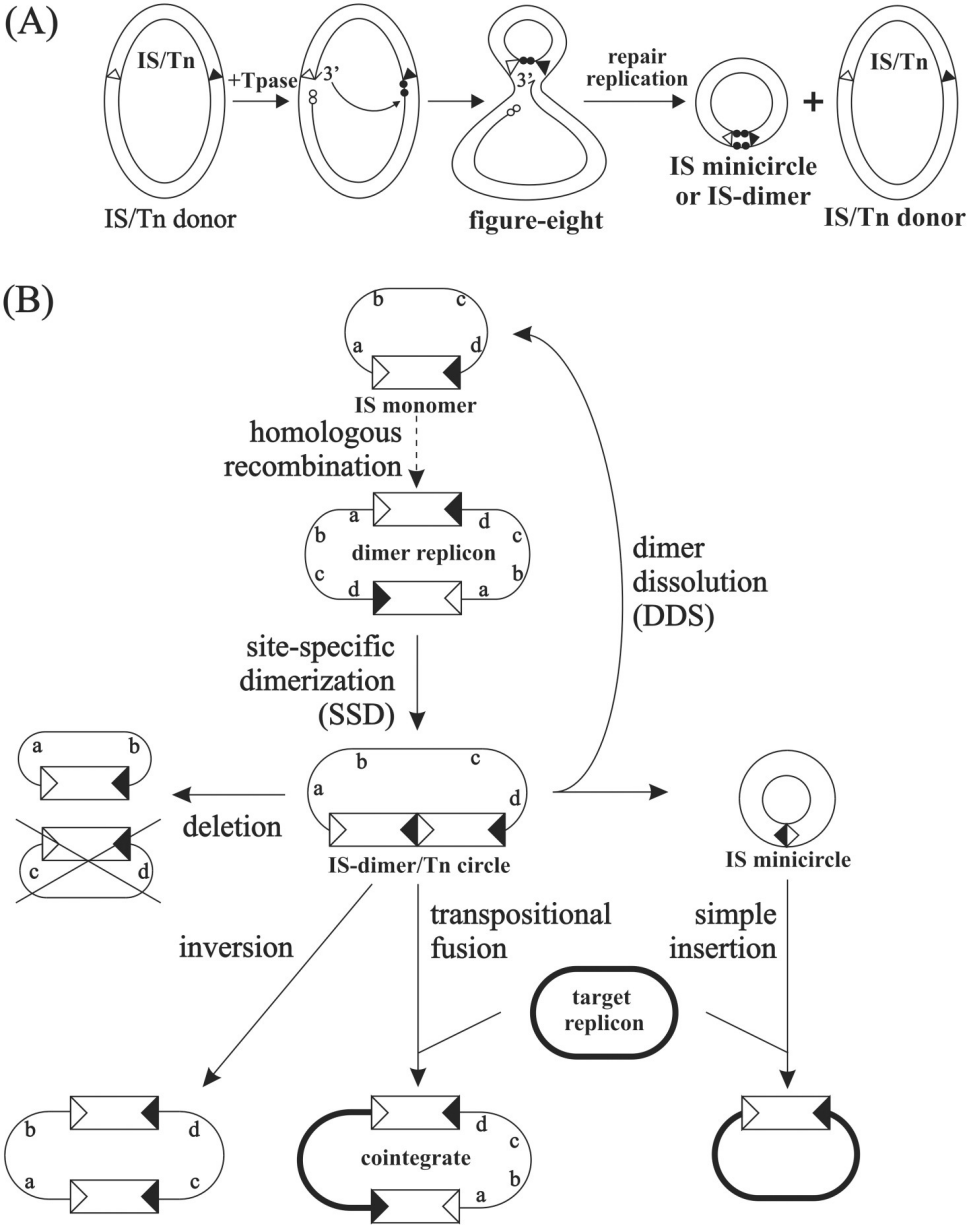
Schematic model of IS*30* transposition. (A) Molecular model of the formation of IS minicircles and IS-dimers (IS*30*)_2_. The IS element is delimited by open (IRL) and filled (IRR) triangles representing the 26-bp left and right inverted repeats, respectively. The two bases adjacent to the Tpase-generated nick next to the donor end are indicated by open circles. The two bases bordering the targeted end at the same strand are shown as filled circles. The free 3’OH ends (3’) are indicated. Note that the same reaction produces a Tn-circle that is formally equivalent to the (IS*30*)_2_ dimer, if the IRs of two IS*30* copies are joined. (B) A mechanistic model of the two-step copy-out-paste-in transposition of IS*30*. The IS elements are shown as open boxes delimited by IRL and IRR. Thin line, donor replicon; thick line, target replicon. Solid and dashed arrows indicate transposition and non-transposition events, respectively. Dimer replicon may arise by homologous recombination. Letters show the order of genes and indicate inversion or deletion on the donor replicon (intramolecular transposition). One of the deletion products is not detectable if it cannot replicate (crossed). IS minicircle is frequently produced from a dimer via DDS, but any IS copy can also produce circular element via a SSD-like process.

The *E*. *coli* element IS*30* applies the copy-out-paste-in mechanism [15] that has also been adopted by members of eight large IS families (IS*1*, IS*3*, IS*21*, IS*30*, IS*256*, IS*110*, IS*Lre2*, IS*L3*) and appears one of the most prevalent transposition strategies among bacterial ISs using DDE Tpases (or DEDD in case of IS*110*-family) [14]. The two major steps of this transposition strategy can be separated in space and time. In the initial step, IS*30* Tpase cleaves one strand at either IS end creating a 3’OH, which, as a nucleophile group, attacks the same strand at the other IS end forming a 2-base single-strand bridge between the tips of the inverted repeats (IR) of the element [16]. This reaction can be considered as a one-ended site-specific transposition. The resulting intermediate, the so-called figure-eight, is processed independently of Tpase by the host replication and repair machinery [17]. The subsequent semi-conservative replication primed by the liberated 3’OH in the DNA flanking the targeted end gives rise to the unaltered donor molecule and the circular transposition intermediate (that was copied out), in which the left and right inverted repeats (IRL and IRR, respectively) are abutted via a 2-bp spacing. This mechanism can account for the formation of both IS*30* minicircles, when the single-strand bridge in figure-eight is formed between the ends of a single IS copy, and IS*30* dimers (signed as (IS*30*)_2_), when IRs of two IS copies are linked [1, 18, 19] (Fig 1A). The latter case is referred as site-specific dimerization (SSD), which can occur on any replicons carrying directly oriented IS copies or a IS*30*-based compound transposon. (IS*30*)_2_ can probably arise also from a SSD taking place on partially replicated plasmids or chromosome, when the replication fork has left a duplicated IS*30* copy, or on dimeric replicons created by homologous recombination of plasmid copies carrying even a single IS [18]. Both types of circular intermediates have an IR-IR junction, which is the substrate of the second transposition step, the integration into the target DNA. While IS or Tn minicircles are non-replicable species whose only chance to be vertically transmitted is the integration into a replicon, IS-dimers can be formed on any type of replicons allowing its propagation and distribution in the bacterial population before integration (Fig 1B).

For integration, two distinct types of preferred target sites can be selected: the so-called hot spot (HS) sequences and the IRs of IS*30* [20, 21]. During integration into a HS site (HS-targeting) Tpase cleaves both strands of the circular intermediates adjacent to the tip of joined IRs and the deliberated 3’OH ends are used to attack the target DNA strands at 2 bp distance [22]. The molecular mechanism of the process is probably similar to that described for ‘non-targeted’ insertion of IS*911* [23, 24]. The remaining gaps and mismatched bases at the IR-HS junctions are processed by host repair leading to the formation of the characteristic 2-bp direct repeats (target duplication) bracketing the inserted element. This way of transposition accompanies the resolution of the active IR-IR junction. In contrast, integration next to an IS*30* IR (IR-targeting) appears to be different. The sequence conversion products arising in IR-targeting events suggest the formation of a branched molecule species by single strand transfer next to the targeted IR and occasional branch migration following gap repair (a nicked Holliday junction is possibly converted into a full Holliday structure) [22]. The molecular steps are probably similar to that of IS*911* ‘targeted’ transposition [25, 26]. Important features of IR-targeting events are the formation of a new IR-IR junction between the targeted IR and one of the donor IRs and the pronounced orientation specificity resulting in exclusively IRL-IRR junctions [1, 18, 21, 27]. A high frequency intramolecular IR-targeting event is when the active IR-IR junction of an IS-dimer reacts with one of the outer IRs of the IS-dimer, leading to the excision of a single element from the dimer. Such excision and, in general, excision of any DNA segments delimited by IS*30* IRL and IRR from an IR-IR junction-bearing replicon is referred as dimer dissolution (DDS) [19]. Both products of DDS (i.e. the donor backbone with the remaining single element and the excised IS or IS-like segment) are circular [22].

Although the integration of a minicircle and an IS-dimer is essentially the same conservative transposition process (paste in), minicircle integration accounts only for simple IS insertions, while the latter results in a cointegrate-like fusion product containing at least two intact elements, thus it is a potential input of further rearrangements. Once formed, the cointegrate can undergo replication, so it can spread in the host population before disintegration. Due to the two directly oriented IS*30* copies, cointegrates can undergo SSD. The further intra- and intermolecular transposition of the resulting IS-dimer can account for creating the classical transposition products, e.g. deletion, inversion and cointegrates (Fig 1B) [18]. This feature might assign important role to cointegrates in promoting cascades of different transposition rearrangements.

The copy-out-paste-in transposition is widely distributed among IS-families [14, 15] and the existence of IS-dimers as transposition intermediates have also been demonstrated for several ISs in the last decades (IS*21* [28], IS*30* [1], IS*2* [29], IS*3*, IS*150* [30], IS*1470* [31], IS*911* [32], IS*256* [33] and IS*1655* [34]. However, the temporal dynamics of transposition rearrangements in the bacterial population, the fate of different intermediates or rearranged products and their contribution to the genetic diversity are poorly explored. Most studies focus on the steps of intermediate-formation and simple insertion or cointegrate formation and further fate of the rearranged products is rarely analysed. In the present work we investigated the products of IS*30*-mediated transposition and their fate both in a plasmid model system and on the chromosome. Taking into consideration of many previous observations and the results of this study, a dynamic model has been proposed for IS*30* transposition. This integrates the known transposition pathways into a complex network that may apply also for other elements that have adopted similar transposition mechanism. The model might give deeper insights into how ISs generate diversity and play a role in retaining the original genetic constitution, whereby they contribute to adaptability and evolution of bacterial populations.

## Results

### Analysis of the fate of IS*30*-mediated cointegrates

**Fig 2:**
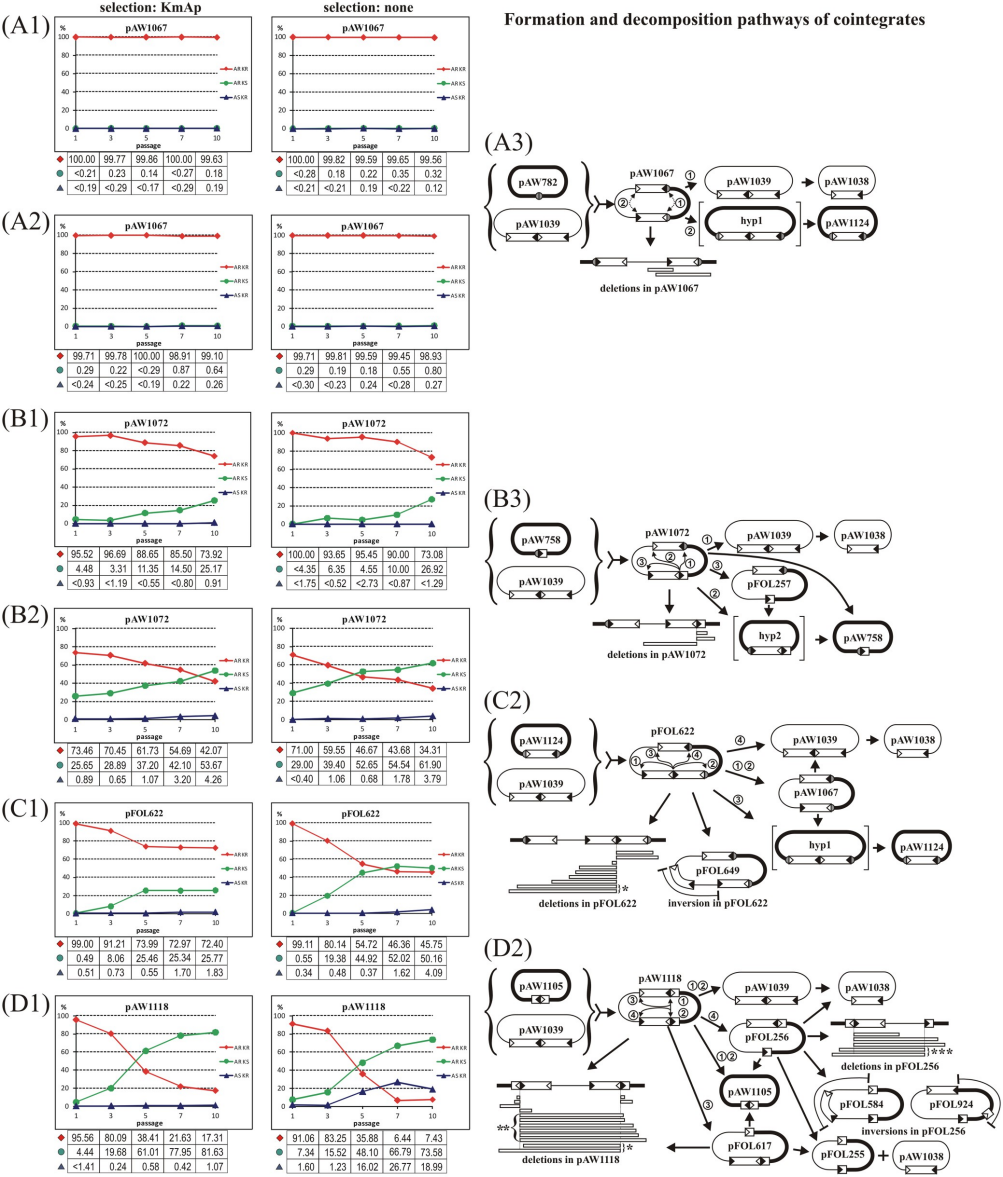
Dynamics and pathways of IS*30*-mediated decomposition of cointegrates. The fate of cointegrates were analysed in *recA*^-^ hosts (TG2; panels A1, B1, C1, D1 and JM109; panels A2, B2) through approx. 50 generations with or without antibiotic selection for the plasmid markers. The relative frequencies of different phenotypes are shown on the graphs and the percent values are given underneath. The three phenotypes are marked as AR KR, AR KS and AS KR (Ap^R^Km^R^, Ap^R^Km^S^ and Ap^S^Km^R^, respectively). Panels A3, B3, C2 and D2 summarize the formation (the parental plasmids of the cointegrates are in curly brackets) and the main decomposition paths of the cointegrates observed in the different hosts and growth conditions. Thin line—p15A based Km^R^ plasmid or part of the cointegrates, thick line–pMB1 based Ap^R^ plasmid or part of the cointegrates. The LHS hot spot sequence and its two halves interrupted by IS*30* elements are shown by shaded circle or semicircles, respectively. Dashed arrows show the alternative SSD reactions in pAW1067. Thin arrows indicate the potential IR-targeting reactions in the original cointegrates, the circled numbers indicate the IR-targeting events and their results. Bracketed structures (designated as hypothetical–‘hyp’) were not isolated as individual plasmid species probably due to their low abundance or stability. Open rectangles below the linear plasmid maps represent different deletions identified (not in scale). Approximate size and the endpoints of deletions were determined by restriction mapping. Inverted regions are indicated, other symbols are as in Fig 1. (A) Decomposition of cointegrate pAW1067 in TG2 (A1) and JM109 (A2) host. (B) Decomposition of cointegrate pAW1072 in TG2 (B1) and JM109 (B2) host. Derivatives of pAW1072 are shown in panel B3. (C) Decomposition of cointegrate pFOL622. The pathways of rearrangements and the resulting plasmid species are summarized in panel C2. The two largest deletions isolated (marked by asterisk) could derive from pFOL622 or the undetected high copy intermediate (hyp 1). (D) Decomposition of cointegrate pAW1118. The plasmid derivatives are shown in (panel D2). Large deletions affecting both IR-IR junctions could derive from pAW1118, pFOL617 (*) or pFOL256 (**). The deletion products of pFOL256 marked with *** could arise in consecutive steps: regeneration of pAW1105 followed by targeting of backbone sequences by the new IR-IR junction. Since pAW1105 cannot code for Tpase, this reaction requires the enzyme synthesized from another plasmids or chromosomal IS*30* copies [35].

Cointegrates carrying at least two IS*30* copies are possibly not the end products of transposition and studying their rearrangements can help to complete our knowledge on the network of IS*30* transposition pathways. Therefore, the fate and rearrangements of four types of IS*30*-generated plasmid cointegrates were first assessed. The cointegrates derived from integration of the (IS*30*)_2_-dimer-containing p15A-based donor plasmid pAW1039 into the compatible pMB1-based target plasmids containing one of the four target types representing all the preferred IS*30* target sites: a natural IS*30* insertion hot spot, LHS [20] representing the nearly palindromic non-IR target sites, an incomplete and an intact IS*30* copy embedded in the LHS sequence corresponding to the possible IR target sites, and an IRL-IRR junction. Thus, the respective cointegrates, pAW1067, pAW1072, pFOL622 and pAW1118 (Fig 2) differed only in the target sequences flanking the IS*30* ends at the donor-target junctions formed during the integration and covered all the genetic contexts in which an IS*30* copy can occur. The four cointegrates were transformed into the *recA*^-^ TG2 and JM109 cells and their fate was monitored through ten passages (50 generations) under selective (LB+Km+Ap) and nonselective (LB) conditions. The *recA*^-^ background prevented the resolution of cointegrates by homologous recombination but had no significant effect on the transposition reactions [18]. Plasmid DNA was isolated from every second passages and transformed into TG2 cells to sort out the unaltered cointegrates and the rearranged products as individual clones. The stability of cointegrates was tested by determining the frequency of plasmid derivatives possessing only one or the other antibiotic markers (Fig 2). Since all cointegrates carried at least two intact IS*30* copies expressing the Tpase and the pMB1 replicon ensured high copy-number, this setup guaranteed a higher Tpase concentration, which facilitated the detection of rare events in the bacterial population as well.

#### Stability of the cointegrates

Plasmid pAW1067, where the fusion of the parental plasmids occurred in the non-IR hot spot sequence LHS, appeared almost stable in both host strains (Fig 2A1 and 2A2) as only few Ap^R^Km^S^ or Ap^S^Km^R^ derivatives were found among approx. 800–1300 colonies examined by replica plating in each passage. In contrast, decomposition of the cointegrates pAW1072 and pFOL622, where one of the donor-derived IS*30* copies was flanked by the LHS as in pAW1067, but the other copy was joined to an IR (Fig 2B3 and 2C2), accelerated (Fig 2B1, 2B2 and 2C1) significantly compared to that of pAW1067. The decay of pAW1118, where both IS*30* copies formed IR-IR junctions (Fig 2D2) appeared the most rapid process (Fig 2D1). Decay of pAW1072 was much faster in JM109 than in TG2 host (Fig 2B1 and 2B2) under both growth conditions, while pAW1118 could even not be maintained in JM109 as the frequency of the Km^R^Ap^R^ transformants dropped below 30% at the first passage. Thus, the fate of pAW1118 and the similarly unstable pFOL622 were examined only in TG2 cells. Omitting the antibiotic selection had no striking effect on the rearrangements, however the decay of pFOL622 and pAW1118 was somewhat faster under nonselective circumstances. Note that all cells remain Ap^R^Km^R^, which still retain even a single copy of the original cointegrate or both of the compatible p15A (Km^R^) and pMB1 (Ap^R^) derivatives of it. It should also be noted that this assay could provide only a rough picture on the fate of cointegrates as the observed frequencies are distorted by the different replication rates of derivatives of the cointegrates and are not directly proportional to the frequency of the primary transposition events. For example, the replication of any p15A-based (Km^R^) derivatives is practically repressed in the presence of the cointegrate, of which copy number is controlled by the high-copy pMB1-based replicon.

For more detailed information regarding the fate of cointegrates, plasmid DNA of individual transformants obtained from each phenotype categories was analysed in every second passages of the two growth conditions. The restriction profile of plasmid DNA samples revealed that the rearranged products were basically similar under selective and nonselective circumstances in both host strains, but their amount and variability depended mostly on the parental cointegrate. In general, the more IRs or IR-IR joints were present in a cointegrate, the more types of daughter plasmids were obtained.

#### Rearrangements of a cointegrate lacking IR-IR junction

As it was mentioned above, rearranged derivatives of pAW1067 were sporadically detected and their temporal accumulation was not significant (Fig 2A1 and 2A2, S1 Table in S1 File). The analysis of plasmid DNA obtained after transformation of the DNA populations isolated from the passaged cultures revealed that the Ap^S^Km^R^ transformants predominantly harboured pAW1038, containing a single IS*30* copy in the intact donor backbone of pAW1039. In several samples pAW1039 and pAW1038 were present together in the mixed plasmid populations. As it was previously shown [18], pAW1039 is unstable and frequently produces pAW1038 by excision of either IS*30* copy (DDS). Therefore, these transformants indicated the presence of pAW1039 in the original DNA populations. On the other hand, the Ap^R^Km^S^ derivatives proved mainly to be pAW1124, the high-copy counterpart of pAW1038 (Fig 2A3). The main path of the rearrangements of pAW1067 thus could be deduced as a two-step process: First, the IS copies of the cointegrate formed an (IS*30*)_2_ dimer (SSD), which restored to the parental plasmid pAW1039 (① in Fig 2A3), then the excision of one IS*30* copy from the IS-dimer (DDS) resulted in pAW1038. A symmetric process could produce the Ap^R^Km^S^ high-copy plasmid pAW1124, an analogue of pAW1038. In this case the SSD reaction occurred between the other two IRs of the IS*30* copies (② in Fig 2A3). Although the (IS*30*)_2_ containing high-copy derivative (hyp1) could not be isolated, pAW1124 probably arose by DDS from this species. The symmetric feature of the decomposition of a pAW1067-like cointegrate had previously been observed with a similar cointegrate, pAW440, where both backbones were p15A-derivative marked with different resistance genes. Decay of pAW440 produced the Km^R^ pAW1039 and pAW1038 and their Cm^R^ equivalents, as well [18]. This result supports that hyp1 also had to be formed, but its extremely rapid conversion into the stable pAW1124 made it undetectable (its instability may be due to the high levels of transposase expressed from P_junc_ promoter formed in the IRL-IRR junction [1] from the high-copy plasmid and the elevated mutation load).

Among the Ap^R^Km^S^ derivatives, several other plasmid species were also found, where a deletion partially or entirely removed one of the IS copies along with its flanking regions. Common feature of these deletions was that they appeared not to be the result of transpositional rearrangements, as the deletion endpoints were distant to the IR ends. The fact that the appearance of the parental target plasmid, pAW782, was not detected among the Ap^R^Km^S^ derivatives of pAW1067 correlated with the earlier observation that the sequence separating the reacting IRs is not released as a circular molecule during SSD and supported that SSD occurs via the figure-eight intermediate [16, 19, 36] (Fig 1A).

#### The effect of an IR-IR junction on the decay of a cointegrate

In the course of passages, the accumulation of rearranged derivatives of pAW1072 containing an IR-IR junction of an intact and a truncated IS*30* copy was faster than those of pAW1067, especially in JM109 host (Fig 2A1, 2A2, 2B1 and 2B2, S1 Table in S1 File). The abundance of the unaltered pAW1072 decreased significantly in both hosts at the 10^th^ passage with or without selection, however, this decrease was more robust under nonselective conditions. In the Ap^R^Km^R^ phenotype category, the cointegrate-like pFOL257 deriving from a DDS achieved considerable proportion with its 13–20% (in TG2) and 7% (in JM109) frequencies. The main segregation product was pAW758 (Fig 2B3), which predominated the plasmid populations at the 10^th^ passage of JM109 host either with or without selection (51% and 59%, respectively) and represented the Ap^R^Km^S^ phenotype practically alone. Similar tendencies were observed with TG2 host, but with lower segregation rates. The general, but low-frequency occurrence of pAW1039 in most passages–considering its short lifetime–suggested its more frequent formation, than in the case of pAW1067 (Fig 2A2 and 2B2). The presence of an IR-IR junction in pAW1072 made possible different segregation patterns, e.g., both parental plasmids of the cointegrate reappeared (compare Fig 2A3 and 2B3). The active IR-IR junction could react with the IRs of the single IS copy and with the outer end of the IS copy forming the IR-IR junction (①-③ in Fig 2B3). Considering that the molecular mechanism of IR-IR junction formation via IR-targeting yields two circular products in intramolecular reaction [22], targeting of the IRR-LHS junction of the single IS*30* copy (①) could reproduce both parental plasmids at the same time (this way can be regarded as the reverse process of integration). The high abundance of pAW758 and the frequent occurrence of pAW1039 in most passages support that this was a preferred reaction. Similarly, targeting the IRL of the same element (②) could simultaneously generate pAW1038 and a hypothetical high-copy plasmid (hyp2) with a complete element joined to the truncated one. However, this plasmid was not directly detectable due to its instability, it could also produce pAW758 via a subsequent DDS reaction. On the other hand, targeting the outer IRR of the intact element forming the original IR-IR junction in pAW1072 (③) could lead to the loss of this IS*30* copy, generating pFOL257. This cointegrate-like plasmid could undergo SSD and a consecutive DDS reaction, which might also produce pAW758 through the hyp2 intermediate. This pathway is virtually identical to that producing pAW1124 from pAW1067 (Fig 2A3). In addition to the IR-targeting events, pAW1072 also produced several Ap^R^Km^R^ and Ap^R^Km^S^ deletion derivatives via intramolecular HS-targeting reactions, which, in contrast to pAW1067-derivatives, arose from transposition as each deletion started at the edge of one IR of the junction (Fig 2B3).

#### Increasing number of IRs opens new decomposition pathways

pFOL622 is similar to pAW1072, except that the IR-IR junction is formed by two complete elements providing one more IR end that can take part in rearrangements. The greater number of possibilities for IR-targeting events (when the active IR-IR junction reacts with one of the available IRs) clearly increased the number of alternative rearrangements, which can explain the faster decay of pFOL622 observed even in TG2 host (compare Fig 2C1 and 2B1). The most prevalent Ap^R^Km^R^ derivative was pAW1067 (Fig 2C2) whose contribution to the whole plasmid population showed an increasing tendency during the 10 passages independently of the selection. pAW1067 could arise through a DDS reaction involving the outer IRs of the (IS*30*)_2_ dimer (Fig 2C2 ①,②) leading to the loss of one IS copy from the dimer. On the other hand, the reactions with IRs of the single IS copy could produce either pAW1039+pAW1124 or pAW1038+hyp1, depending on which IR was targeted (Fig 2C2 ③,④). Furthermore, pAW1038 and pAW1124 could also arise through further decomposition steps of pAW1067 as described previously (Fig 2A3). Not surprisingly, pAW1124, which has advantages in propagation due to its alternative ways of formation, high copy number and elevated stability because of its reduced IS content, was the other most abundant product. Plasmids pAW1038 and pAW1039 were also detectable in nearly every passage, despite that their replication was probably almost completely inhibited by the multicopy cointegrates, which indicates that targeting of the IRR-LHS junction in pFOL622 (④) was a relatively frequent reaction.

pFOL622 proved also to be more active in non-IR-targeted rearrangements than pAW1072. In the deletion products and the inversion derivative, pFOL649, the plasmid backbone or inner sequences of IS*30* copies were targeted. These rearrangements, as observed previously with pAW1072, were always initiated by the IR-IR junction and led to its elimination. The deletion and inversion products together represented an increasing portion of the Ap^R^Km^S^ derivatives during the passages, probably due to their higher stability compared to pFOL622.

#### The activity of two IR-IR junctions yields a broad spectrum of rearrangements

The cointegrate pAW1118 derived from a site-specific reaction between two IR-IR junctions (Fig 2D2) [37]. As it is a preferred event [21], the reverse reaction inside the cointegrate was the main cause of its fast disintegration observed even in TG2 host. The most rapid way of pAW1118 decay accompanied the accumulation of the parental target plasmids pAW1105 and pAW1039 under selective or nonselective conditions, as well. Consequently, the proportion of the unaltered pAW1118 and its Km^R^Ap^R^ derivatives dropped from 96% to 17% under KmAp selection and from 91% to 7% without selection through the 10 passages. The contribution of pFOL256 and pFOL617 (the alternative products of the otherwise frequent IS excision from the IR-IR junctions by DDS) to the Km^R^Ap^R^ plasmid populations remained low under any selection conditions, which emphasized the dominance of the site-specific reaction mentioned above. It should be noted that pFOL256 and pFOL617 are high copy IR-IR junction-bearing species, which might rapidly lose their full IS*30* copy in a second DDS reaction and be converted to the more stable cointegrate-like plasmid pFOL255. Even though this plasmid is expectedly a more stable product (at least in the lack of other Tpase-producing plasmids), its contribution to the plasmid populations was rather low. One explanation can be that both pFOL256 and pFOL617 had an alternative way for further decomposition, i.e., the IR-IR junction could interact with the single truncated element, producing pAW1105 and pAW1038 at the same time. This was supported by the segregation test where pFOL256 was individually assayed (S1 Fig and S1 Table in S1 File). In this case, in addition to the formation of pFOL255, both pAW1105 and pAW1038 were detectable from the 3^rd^ passage under any growth conditions.

As expected, pAW1118 also showed an elevated HS-targeting activity resulting in many different deletion products. Furthermore, besides the deletion derivatives, two inversions were also identified among the products of pFOL256. In these derivatives mostly the plasmid backbone sequences were targeted, and the deletions eliminated the full IS*30* copies, leading to stable products (Fig 2D2). Although their active rearrangements were blocked, they could still serve as targets for further transposition, which might turn their fate back into new rounds of rearrangements.

### Genome rearrangements promoted by IR-IR junctions

**Fig 3:**
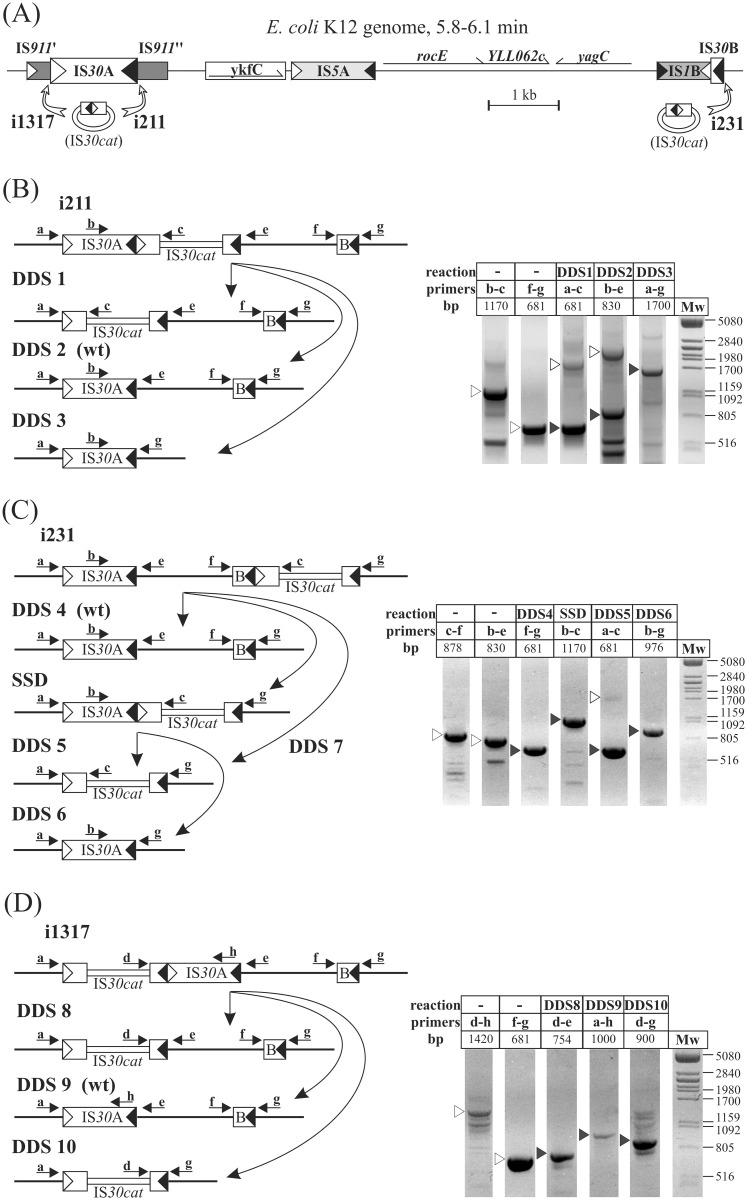
Chromosomal rearrangements promoted by the IR-IR junctions formed by IS*30cat* insertions. (A) The schematic map of the 5.8–6.1 min chromosomal region of *E*. *coli* K-12. The mobile elements or transposon-like sequences are shown as rectangles with different patterns. Open and filled triangles show IRL and IRR of the IS elements, respectively. The IS*911* sequence is incomplete and interrupted by the insertion of IS*30*A (the two parts are indicated as IS*911*’ and IS*911*”). Other ORFs are shown as thin arrows. IS*30*B is a truncated copy including the last 184 bp of the element. The open arrows point to the integration sites where IS*30cat* inserted in the three TG2-derived strains i1317, i211 and i231. The map is based on the chromosome of *E*. *coli* K-12 W3110. (B-D) Genomic rearrangements in the population of descendants of strains i211 (B), i231 (C) and i1317 (D). Left panels show the expected products generated via targeting the IR ends by the active junctions (disregarding the IRs of other two resident IS*30* copies, IS*30*C and IS*30*D, which locates far from this region, consequently, the reaction with their IRs would generate large chromosomal deletions that are most likely lethal for the host cell). The right panels show the products of PCRs designed to detect the original constitution (open arrowheads) or the expected rearrangements (SSD and DDS1-10, filled arrowheads). Lettered arrows show the positions and directions of primers on the graphs. The primer pairs used in PCRs and the expected fragment sizes are shown above the lanes. DDS reactions restoring the genomic state prior to IS*30cat* insertion in each strain are marked as wt. Note that in i231, DDS5 and DDS7 lead to the same product. For uncropped images, see S2B-S2D Fig in S1 File.

The previous analysis of the fate of different cointegrates revealed how dual target specificity of IS*30* leads to an enormous diversity of descendant plasmids in a bacterial population. Since the rearrangements of plasmids are generally not as restricted by the selective pressure as those of the chromosome, we decided to study whether the activity of an IS*30* dimer located on the bacterial chromosome has similar consequences. We have previously isolated *E*. *coli* strains carrying an extra IS*30* element labelled with Cm^R^ marker gene (IS*30cat*) on their chromosomes [19]. These strains were generated by chromosomal insertion of IS*30cat* minicircles established *in vitro*. Since the minicircles did not carry intact Tpase gene, the integration was promoted by expression of IS*30* Tpase *in trans* from the producer plasmid pJKI132 [19], which was still present in the insertion strains used in this experiment. The increased level of Tpase due to the leaking expression from the tac promoter of pJKI132 helped to reach higher transposition activity, which facilitated the detection of rear events. In three strains, the IS*30cat* element inserted next to the IRs of IS*30*A and IS*30*B, the resident IS*30* copies in *E*. *coli* K-12 [38] (Fig 3A). Therefore, these strains carried an active IR-IR junction on their chromosome. For comparison, two further strains, in which IS*30cat* element inserted at non-IR (e.g. HS) sequences (i232 and i1313) in different chromosomal positions [19] were also examined.

#### Analysis of chromosomal rearrangements

First, the stability of insertions was investigated. The original stocks of i211, i231 and i1317 strains kept on –70°C were plated and grown overnight under Cm selection. The obtained colonies were tested by colony PCR for the presence of the original IR-IR junction. The result was positive in 7 out of 28 colonies for i211 and 3 out of 15 colonies for i231. Even though the PCR proved the presence of the junction in all three original stocks, no junction was detected even in 227 progeny colonies of i1317. The extreme instability of this insertion can be explained by the configuration of the IS*30cat*-IS*30*A dimer, forming a strong P_junc_ promoter [1], which could produce high levels of Tpase from ORF-A of IS*30*A. In strains i211 and i231, the same promoter is directed toward the IS*30cat* element where ORF-A is missing. Three IR-IR junction-bearing colonies from i211, i231 and 3 colonies of strains i232 and i1313 (lacking chromosomal IR-IR junction) were grown under nonselective conditions to examine the stability of IS*30cat* element. Both i211 and i231 derivatives showed high frequency loss of IS*30cat* as the mean rate of Cm^S^ colonies was 13.8±7.8% or 39.8±4.4%, respectively. Conversely, the IS*30cat* element of i232 and i1313 proved stable (Cm^S^ was not detected among approx. 1000 colonies, their mean frequency was <0.33±0.05%).

To analyse the rearrangements, total DNA was isolated from the original stock of i211, i231 and i1317, representing the population of immediate descendants of cells where IS*30cat* insertion occurred. If the active junction promotes rearrangements on similar pathways as did in the plasmid-borne systems, the end products are predictable. Supposing that the preferred reaction is the DDS (i.e., the excision of any “IS-like” DNA segment that is bordered by a left and a right IR taking part in an IR-IR junction) as observed previously, the original constitution in i211 can be resolved by three ways (Fig 3B). In addition to the original IR-IR junction, all the expected products were detectable by appropriately designed PCRs in the same DNA population (Fig 3B, right panel). More complex network was anticipated in case of i231 as not only one-step DDS, but the formation of a new dimer by SSD was also possible (Fig 3C) and the resulting new (IS*30*)_2_ had two additional ways to be resolved. The PCR assay proved that all the predicted structures were present in the DNA population deriving from i231 strain, likewise in i1317, where three alternative DDS reactions were expected and verified (Fig 3D). The IR-IR junction-mediated rearrangements can also be directed towards non-IR HS sequences. These events (mostly deletions) might be represented by the minor PCR-products (Fig 3B–3D, right panels). The fact that all the predicted IR-targeted reactions and many other rearranged species were detectable strengthened that the rearrangements observed in the plasmid cointegrate system properly represent the events at genomic scale.

#### The IR-IR junction is an efficient generator of genetic variability and acts as a powerful mutagen

**Fig 4:**
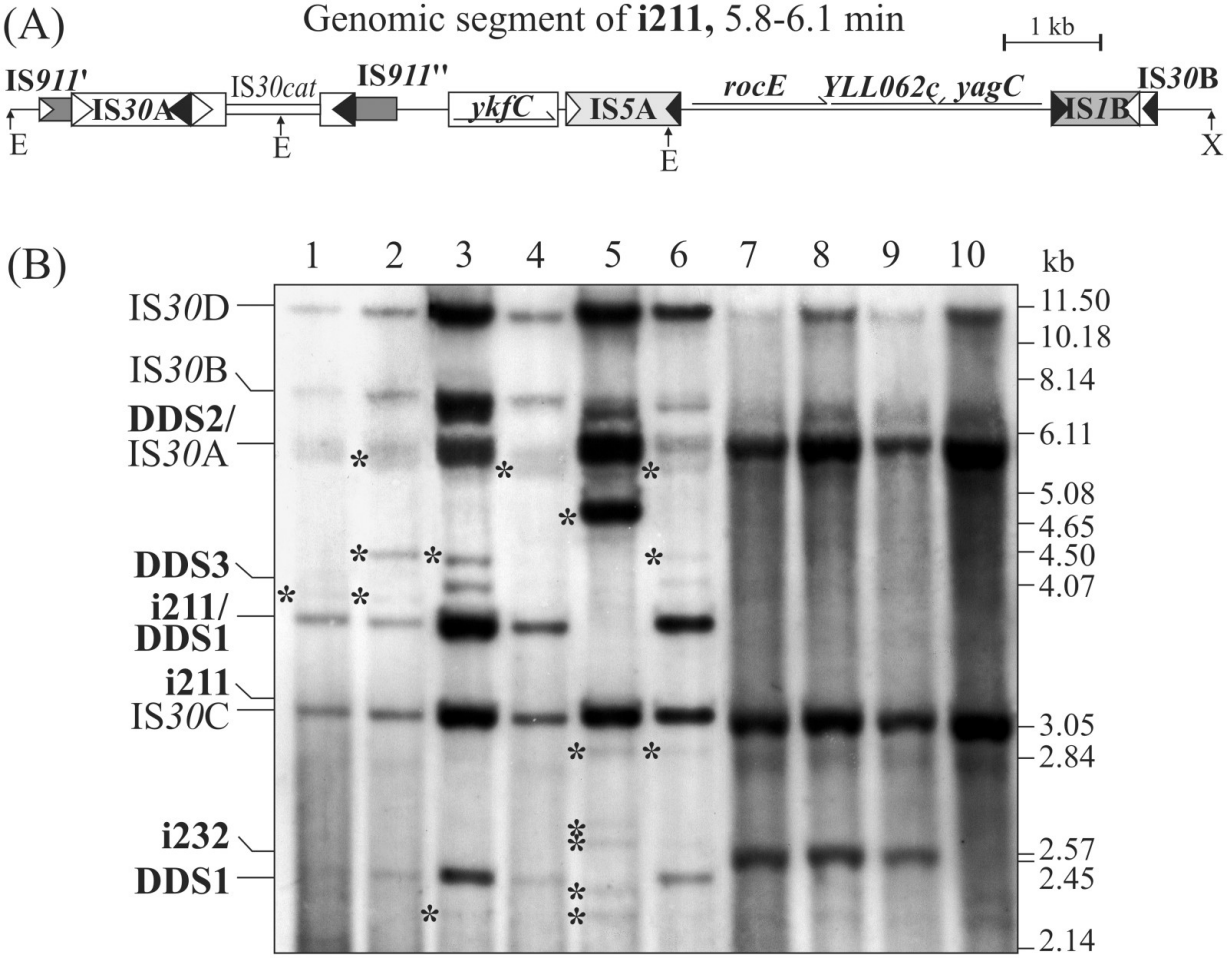
Southern analysis of rearrangements in i211 and i232 insertion strains. (A) The schematic map of the 5.8–6.1 min genomic region of *E*. *coli* TG2 derived strain i211, where IS*30cat* has inserted next to the IRR of IS*30*A. Symbols are as in Fig 3A. (B) A representative set of genomic DNA samples of individual clones derived from the descendants of i211 (lanes 1–6) and i232 (lanes 7–9). Lane 10 –total DNA of strain TG2. The total DNA digested with *Eco*RI-*Xho*I (E and X indicates the respective restriction sites on panel A) were hybridized to a full length IS*30* probe. Bands representing the resident IS*30* copies are indicated as IS*30*A-D, while those referring to the original insertion or the expected DDS derivatives are in bold. Unidentified hybridizing bands (marked by asterisks) may represent insertions, deletions, or inversions. In contrast, i232, where the IS*30cat* insertion occurred at a chromosomal hot spot (lanes 7–9) did not produce any rearranged products. For uncropped image, see S3 Fig in S1 File.

The additional minor PCR fragments not corresponding to any products of the expected transposition reactions suggested that many HS-targeting events occurred in the primary bacterial populations. To study some of these events, an aliquot of the original i211 stock (for the chromosomal state see Fig 4A) was overnight grown under selective (LB+Cm+Ap) and nonselective (LB+Ap) conditions and spread on LB+Ap plates. Total DNA of 32 and 38 colonies deriving from growth under CmAp and Ap selection, respectively, was isolated and analysed by Southern hybridization with IS*30*-probe. Remarkable diversity was observed in both sets of samples as not simply the three DDS products (DDS1, 2 or 3, Fig 3B), but many insertions and other rearrangements (e.g., DDS combined with a deletion or insertion) were also detectable. Summarizing the data obtained from the 70 individual colonies (a representative set is shown in Fig 4B, lanes 1–6), 24 DDS1, 6 DDS2, 2 insertions (transposition of IS*30*) and 4 deletions were identified in the CmAp-selected group (two or more rearrangements were observed in 7 colonies), while 24 DDS1, 14 DDS2, 7 DDS3 and 13 deletions were found in the Ap-selected group (one clone was unaltered and two or more rearrangements were detected in 13 colonies). For comparison, in a similar assay, the strain i232 lacking IR-IR junction appeared completely stable showing no additional bands (Fig 4B, lanes 7–9).

The mutagenic effect of the active junction present in the chromosome of i211 and i231 strains was also assayed along with two control strains, i1313 and TG2 (all harboured the inducible Tpase producer plasmid, pJKI132 [35]) by examining the emergence of loss-of-function mutants in maltose, galactose and xylose metabolizing capacity. Sugar non-utilizer mutants appeared for all three sugars with a frequency of 2.0–4.5×10^−4^ in both junction-bearing strains, while no such mutants could be isolated from the control strains lacking active junctions (S2 Table in S1 File). The Southern analysis and these data clearly showed that an IR-IR junction acts as an efficient mutagen, which increases the genetic diversity of the bacterial population.

## Discussion

In the present work we studied the consequences of IS*30* transposition on the fate of various model plasmids and the chromosomal DNA of *E*. *coli*. As many other ISs, IS*30* generates insertions, deletions, inversions, and replicon fusions, which are often regarded as end-products of transposition. Our study demonstrates how these structures re-enter the transposition circuit and maintain a complex transposition network in the population of host bacteria. There are some important features of IS*30* transposition that can account for the observed paths of rearrangements and the variability of the descendant populations. (i) The element transposes via an active structure formed by joining the left and right IRs [18], [19]. (ii) The active junction can be formed via SSD [16] or targeting an IS*30* IR by a structure already possessing an IR-IR junction (insertion of IS*30* minicircle or (IS*30*)_2_ dimer next to an IR, [21, 22]). (iii) IS*30* has a dual target specificity: it can integrate into HS sequences [20] or next to IS*30* IRs [21]. (iv) While the HS-targeting ceases the active IR-IR junction, the IR-targeting yields a new one [20–22].

The rearrangements of plasmid cointegrates and the chromosomal DNA showed essentially the same patterns, thus, the cointegrates proved to be useful models for the genome-scale events. The cointegrate containing sole IS copies (pAW1067) embedded in HS sequences and lacking active IR-IR junction appear quite stable similarly to the sole ISs on the chromosome. In this case the cointegrate shows low level of decomposition and produces few types of derivatives. Aside from the rare non-transposition events, remarkable feature of the rearrangements is that the parental donor plasmid reappeared, but the original target had never been detected among the derivatives by the applied method. This observation supported our earlier findings that precise conservative IS/Tn excision regenerating the original non-IR target site does not occur in IS*30* transposition [18, 19, 36] which agrees well with the prediction of the proposed copy-out-paste-in model (Fig 1A). In contrast, plasmid species carrying a single element inserted in the original target plasmid have emerged (pAW1124, Fig 2A3). This observation is in correlation with the molecular mechanism of junction formation by SSD [16] and the subsequent IS excision from the IS-dimer-containing derivative (which is formally equivalent to a transposon circle, [19]) via DDS (see also Fig 1). Even though the species with single ISs are far more stable than the IS-dimer-bearing structures, they can be the source of IS-minicircles by SSD [19] and serve as targets for IR-targeting (see the formation of pFOL622, Fig 2C2). The results support that the “classical” cointegrate is not an endpoint of transposition, it is rather a source of new plasmid species even though the low rate of its rearrangements. Similar stability and low transposition activity was observed with the chromosomal single IS*30cat* insertions where no genome scale rearrangements were observed among the analysed clones [19], and the mutation frequency was below the detection limit even though a higher Tpase concentration due to the presence of a Tpase producer plasmid (see strain i1313 in S1 Table in S1 File).

The presence of an IR-IR junction strikingly stimulates the decomposition of cointegrates. Accordingly, the increasing number of available IRs to be targeted opens more transposition pathways and gives rise to wider spectrum of derivatives (Fig 2B3, 2C2 and 2D2). Each cointegrate formed by IR-targeting could reproduce both of its parental plasmids through the “reverse” of the original IR-targeting reaction or through several alternative IR-targeting steps. These reactions produce new IR-IR junctions, and all are reversible.

Similar processes have been observed with the genomic IS*30cat* insertions forming chromosomal IS-dimers. *E*. *coli* K-12 harbours an incomplete and three intact IS*30* copies [38], and the integration of a new element next to the IR of any one generates an IR-IR junction on the chromosome. Targeting of IRs at the 6 min chromosomal region where IS*30*A and the truncated IS*30*B reside (Fig 3A) results in analogous structures to the cointegrates derived by IR-targeting (Figs 2B3, 2C2, 2D2 and 3A). Each chromosomal IR-IR junction-initiated cascades of rearrangements including IR- and HS-targeting events (Figs 3B–3D and 4B). The junctions were resolved through similar pathways that were observed in the plasmid-based system. All expected products of the possible DDS and SSD reactions could be detected in the same bacterial populations along with many different insertions or deletions derived by HS-targeting events (Fig 4). The high frequency of sugar non-utilizer mutants observed in such strains (S2 Table in S1 File) also indicates the efficiency of IR-IR junctions in mutagenesis and generation of genetic diversity in the bacterial population.

**Fig 5:**
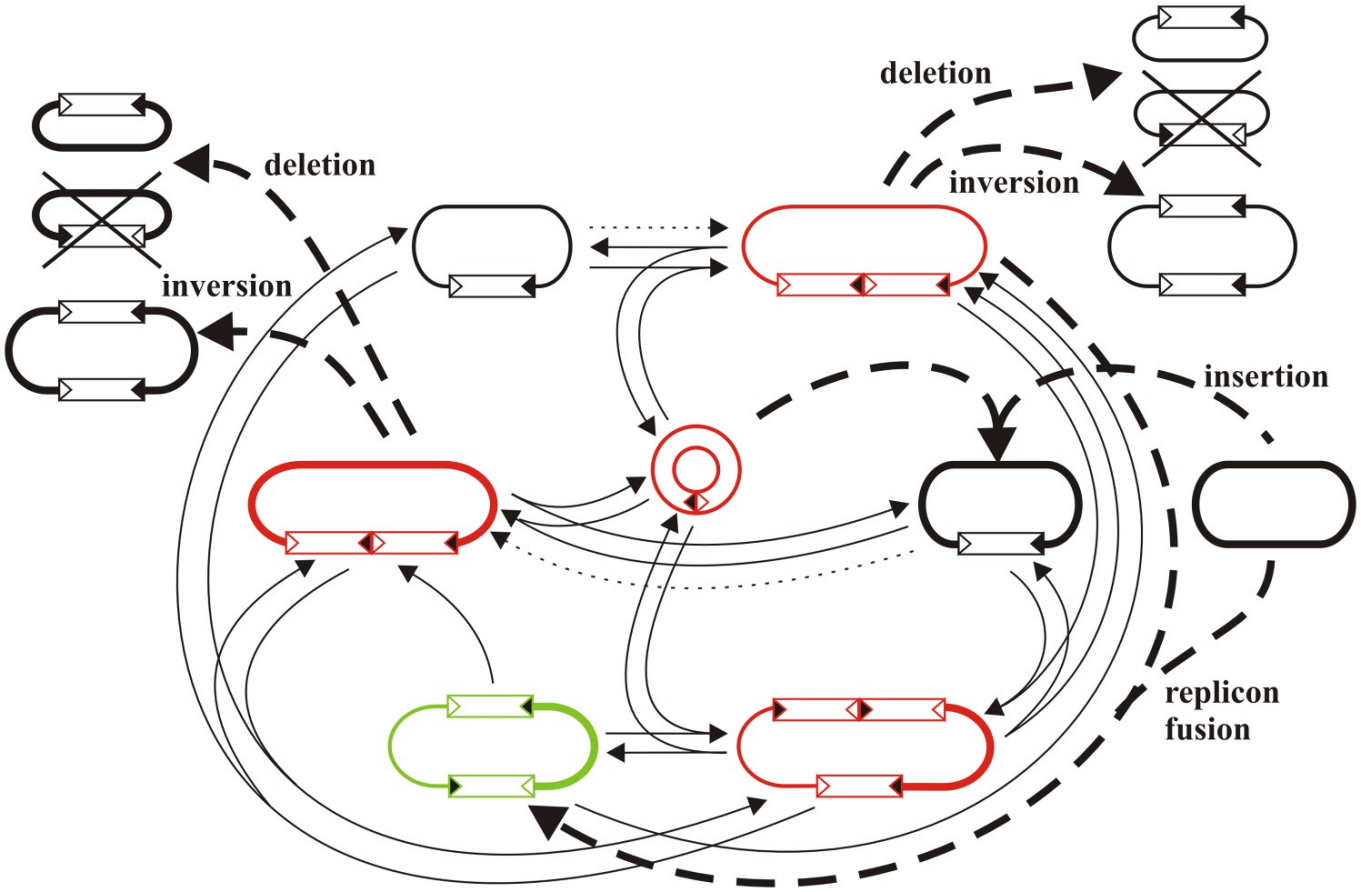
The dynamic model of IS*30* transposition. The model integrates the results of this work and those published previously [18–22]. The reversible IR-targeting events (shown by thin arrows) always produce molecule species that carry an active IR-IR junction (shown as red) and define a closed circuit. HS-targeting events (shown by thick dashed arrows) provide the break-out pathways and produce the “classical” transposition products, like insertion, deletion, inversion, or replicon fusion (shown as black). These rearrangements are irreversible and produce stable molecule species. One of the two deletion products generally has no replication origin, so it is not detectable (crossed out). Dotted arrows represent consecutive steps of a non-transpositional (formation of dimer replicon by homologous recombination or partial replication) and a transpositional (SSD) process resulting in an IS-dimer. Note that minicircles do not only derive as products of DDSs, but can emerge from any IS-containing replicon via an SSD reaction (not indicated) [16, 19]. Apart from minicircles and some deletion products most components of the network can replicate and survive even many cell cycles ensuring that many different plasmid species and/or chromosome can occur in the same bacterial population. The classical cointegrate structure (shown as green) may have an important role in maintaining the network. It is stable enough to spread in the whole population and can produce active structures (IS-dimer) via a single SSD reaction, by which it can be the start point of bursts of rearrangements in different cells in the population.

Taking together our observations from both systems and the knowledge gathered previously on IS*30* transposition, we have to expand our earlier–more or less static and linear–model (Fig 1) to a more dynamic interpretation (Fig 5). In this dynamic network, the IR-targeting events themselves define a closed transpositional circuit where each event resolves and reproduces IR-IR junctions and all reaction steps are reversible. The complexity of the network increases with the number of IR ends available and many molecule species can be regenerated even in alternative ways. One way to break this “endless” circuit is DDS reaction producing nonreplicable IS or Tn minicircles that can be lost if their reintegration does not occur before cell division. The other way is HS-targeting, which resolves the IR-IR joints and leads to the most stable structures like insertion, deletion, or inversion. Although these products can be considered as exit points of the circuit showing low transposition activity, they can still act as targets for IR-targeting and produce minicircles through the figure-eight (Fig 1A) or IS-dimers by SSD during plasmid or chromosome replication. Unlike IR-targeting reactions, HS-targeting is irreversible as deletion or inversion products cannot regenerate their progenitor molecule or a simple insertion cannot be reversed by transpositional excision (the “IS-free” status can be achieved only by the very rare illegitimate recombination [36]).

Theoretically, the transposition network shown in Fig 5 requires two replicons (e.g., a plasmid and a chromosome) and a single IS*30* element as input. A minicircle having an IR-IR junction can arise by SSD through figure-eight processing [19], then it can integrate next to the original copy creating an IS-dimer. Non-transpositional processes like homologous recombination of identical replicons carrying the single element or theta-like replication through the IS copy can yield molecule species that can also be the substrates of SSD reaction, generating IS-dimers. The emergence of an (IS*30*)_2_-containing replicon in a single cell can lead to a cascade of rearrangements due to the replication. Although IS-dimers appear very unstable in high-copy plasmids, they are stable enough in low-copy replicons or the chromosome [19, 39] to spread in the population. Then, the different reactions at different times ensure the generation of many divergent products. It seems clear that the establishment of the entire circuit in a single cell is unlikely, however, it can be maintained for a longer time in the whole population.

The establishment and maintenance of such transposition circuit depends on many factors such as (i) the frequency of IS-dimer and minicircle formation by SSD, (ii) the probability of vertical transmission of an IS-dimer during cell division, (iii) the number and location of IR and HS sequences in the genome, which may influence the choice between IR- or HS-targeting, (iv) the bias between the efficiency of IS-excision from IS-dimers by DDS and the processes creating IS-dimers, which depends on the host recombination, repair and replication machinery [16–19] (v) environmental conditions that influence the bacterial growth and the propagation of the subpopulation carrying different plasmid and chromosome forms (selection).

The lifetime of dimers exceeding the time of a cell cycle or frequent generation of dimer-containing replicons increase the probability of establishing a complex network. On the other hand, if the target choice is shifted towards HS sequences and/or disintegration of dimers via DDS is faster than cell division, the rapid withdrawal of active structures will lead to breakdown of the transposition circuit. Our observations suggest that an IS-dimer formed on a low copy plasmid or the chromosome is stable enough to spread in a detectable portion of a bacterial population (Figs 2 and 3). It is worth to note that the frequencies of IR- and HS-targeting by the minicircles may not differ with orders of magnitude, as both events could be detected among less than 30 genomic insertions analysed [19]. These observations and the results obtained from the decay of cointegrates and the chromosomal IS*30* dimers affirm that the dynamic transposition network can be established and maintained in the bacterial population through at least 50 generations or even 30 years under extreme conditions of stab cultures [39].

The proposed model can explain the bursts of transposition events observed in the strains having genomic IS-dimers formed by IS*30cat* insertions (Fig 4, S2 Table in S1 File) and in stab cultures, where a chromosomal (IS*30*)_2_ dimer emerged [39]. In both cases, even though the quite different circumstances, the chromosomal IS-dimers were stable enough to spread in the populations and generated many different rearrangements. It is questionable how long and at what extent the diversity of a population can be maintained. Stabs where starvation prevents the growth of culture seem to provide better circumstances for accumulation of different variants [40], which was observed in samples collected through 30 years [39, 41]. In contrast, based on the classical view of evolution in asexual organisms [42], one can expect that in a continuously growing culture the fittest phenotype quickly overgrows the others (clonal shift) leading to homogeneous population. However, considerable diversity was observed in cultures grown 10000 generations [7]. We have shown that–under certain conditions–the parental plasmid species as well as the original chromosomal constitution preceding the IS-insertion are restored and survive among the rearranged products. Thus, our model includes “feedback loops” that conserve (i.e., regenerate) the original genetic constitution, while others maintain the active structures causing bursts of rearrangements, which can account for long term maintenance of the original genotype and the diversity of emergent genotypes even under very different circumstances.

Numerous IS families (IS*1*, IS*3*, IS*21*, IS*30*, IS*256*, IS*110*, IS*Lre2*, IS*L3*, [14]) have adopted the copy-out-paste-in transposition strategy involving minicircle intermediates and some ISs proved also to generate IS-dimers (IS*3*-family: IS*2*, IS*3*, IS*150* and IS*911* [29, 30, 32], IS*30*-family: IS*30*, IS*1470* and IS*1655* [1, 31, 34]; IS*21*-family: IS*21* [28]; IS*256*-family: IS*256* [33]). Although dual specificity in target selection has been documented only for IS*911* [25], the capacity of the abovementioned ISs for the formation of minicircles and IS-dimers may refer to similar transposition pathways to that of IS*30* and suggests that our integrated transposition network may apply for them, as well.

## Materials and methods

### Bacterial strains, media, and chemicals

The *recA*^-^ *E*. *coli* K-12 strains JM109 [43] and TG2 [44] were used in the experiments. Bacteria, if other is not indicated, were grown in Luria-Bertani (LB) medium supplemented (if necessary) with 150 μgml^-1^ ampicillin (Ap), 20 μgml^-1^ kanamycin (Km) or 20 μgml^-1^ chloramphenicol (Cm). Common DNA techniques were performed according to [44]. Restriction enzymes and DreamTaq polymerase were purchased from Fermentas (Thermo Fisher Scientific).

### Plasmids

Cointegrates were products of transpositional fusion of the Km^R^ p15A-based donor plasmid, pAW1039 [18], and the Ap^R^ pMB1-based pEMBL19 [45] derivative target plasmids (Fig 2A3, 2B3, 2C2 and 2D2). pAW1067 was formed by integration of pAW1039 into the natural IS*30* hot spot, LHS (identified in the 33244–33498 bp of λ phage), carried by pAW782 [20]. pAW1072 was obtained similarly by fusion with the target plasmid pAW758 [21] containing an IS*30* right end flanked by the left side part of LHS (33244–33290 bp of λ). In the case of pFOL622, the parental target plasmid pAW1124 harboured an intact IS*30* element embedded in the LHS sequence (pAW1124 is the insertion derivative of pAW782, where the IS*30* inserted at the central 2 bp of LHS), while in the case of pAW1118, the parental target plasmid pAW1105 carried the joined left and right ends of IS*30* [21].

### Assay for the rearrangements of cointegrates

The cointegrates were introduced into the *E*. *coli* strains TG2 and JM109 and the transformants were selected on LB+Km+Ap plates. A single colony from each transformation was used to inoculate 3 ml LB and LB+Km+Ap broth and the cultures were grown overnight at 37°C (first passage). 0.1 ml of each culture was transferred into 3 ml of fresh medium (LB or LB+Km+Ap, respectively) and grown overnight. The passage steps representing about 5 generations were repeated 10 times (approx. 50 generations). Plasmid DNA was purified from the 1^st^, 3^rd^, 5^th^, 7^th^ and 10^th^ passages and transformed into TG2 cells. Transformants were selected on LB+Ap and LB+Km plates. The relative frequency of the resistance phenotypes (Km^R^Ap^R^, Km^R^Ap^S^ and Km^S^Ap^R^) was determined by replica plating of the transformants onto LB+Ap and LB+Km plates. Plasmid DNA was purified and analysed by restriction digestions from at least 20 colonies of each phenotype from all the examined passages (except in the cases, when less than 20 colonies were obtained in a particular phenotype category after testing at least 500 colonies by replica plating).

### Analysis of the fate of genomic IS*30cat* insertions

For this analysis, TG2-derivative strains harbouring chromosomal insertions of the modified element, IS*30cat* carrying the Cm^R^ gene of Tn*9*, were used [19]. Strains harbouring chromosomal IR-IR junction were: i211 and i1317, where IS*30cat* was inserted adjacent to the right or the left IR of IS*30*A, respectively, and i231, where the insertion occurred next to the IRR of the truncated IS*30*B (Fig 3A). In two further strains, i232 and i1313, the IS*30cat* inserted into non-IR target sites at approx. 61.9 and 59.4 min of the *E*. *coli* chromosome, respectively (the insertion strains analysed were formerly designated as IGEN211, IGEN1317, IGEN231 and IGEN232 [19]). Additionally, all strains harboured the Ap^R^ inducible Tpase producer plasmid, pJKI132 [35]. ORF-A encoding IS*30* Tpase is placed under the control of *tac* promoter in pJKI132.

For the stability assay, aliquots of the original stocks of the five IS*30cat* insertion strains stored at –70°C were plated and grown overnight under Cm (20μg/ml) selection. The colonies were pre-screened by colony PCR (see below) for the IR-IR junction present in the original strains i211, i1317 and i231 than three parallel colonies that were positive in PCR test were picked and grown overnight in LB+Ap supplemented with 10 μM IPTG to slightly induce Tpase expression. Then the frequency of Cm^S^ cells in the overnight cultures was determined by replica plating.

Total DNA of the original strains i211, i231 and i1317 was used for PCR analyses to detect DDS and SSD reactions. For the amplification the following primers were used (see also Fig 3): **a**, tggaattcggcctttccacaatgacgcg; **b**, tgagacaatttttaaaacgctgt; **c**, agtgataataagcggatgaatgg; **d**, cagctcaccgtctttcattgc; **e**, cggaattcagcccgtctgccgtctgg; **f**, gtgaattccagttgccatgttttacggc; **g**, ttgaattcccacgggtttaacagacacc; **h**, atcgtataacgcgtttttcggtctac. PCR cycling was as 94°C for 1 min, 40 cycles of 94°C for 20 sec, 55°C for 1 min and 72°C for 3 min, followed by 72°C for 5 min.

### Southern analysis

To obtain individual colonies from i211- and i232-derived populations, 10 μl aliquot from the original stocks stored at –70°C was grown overnight at 37°C in 5 ml LB+Cm+Ap broth. 200 μl of the cultures was transferred into 5 ml LB+Ap or LB+Cm+Ap supplemented with 10 μM IPTG and incubated 20 hours at 37°C. Single colonies were isolated by plating the cultures on LB+Ap and their resistance phenotype was determined by replica plating. Total DNA from 38 i211 and 32 i232 derivative colonies was isolated and analysed by Southern hybridization using the whole IS*30* sequence as a probe. Total DNA was overnight digested with *Eco*RI-*Xho*I and separated on 0.8% agarose gel for 20 hours at 4°C and 15 mA. Hybridization and labelling were performed using the DIG DNA Labelling and Detection Kit (Roche) according to the manufacturer’s recommendations. Membranes (Biodyne) were developed using the fluorescent substrate CSPD (Roche) and exposed onto Kodak X-OMAT film.

### Assay for the mutation rate in sugar-metabolism

Three single colonies from i211, i231, i1313 and TG2 strains containing also the Tpase producer plasmid, pJKI132 were grown overnight at 37°C in 2 ml LB+Ap broth. The presence of the original IRL-IRR junction in the selected colonies from i211 and i231 were proven by colony PCR using primers **b-c** and **f-c**, respectively (Fig 3B and 3C). The overnight cultures were spread onto EMB agar plates [46] supplemented with 1% of maltose, galactose or xylose. White and pale pink colonies were individually tested on EMB+sugar, M9+sugar, LB+Ap and LB+Cm agar plates. The colonies that were colourless again on EMB+sugar and not viable on M9+sugar, were considered as sugar non-utilizer mutants.

## Supporting information

S1 File Contains all the supporting figures and tables (including S1-S3 Figs and S1, S2 Tables). (PDF)

S1 Raw images (PDF)
