# Supplementary material for: The dynamic network of IS30 transposition pathways
Source: PLoS One. 2022 Jul 28;17(7):e0271414. doi: 10.1371/journal.pone.0271414 (PMC9333248; doi:10.1371/journal.pone.0271414)
Supplement: S1 File — (PDF) [file pone.0271414.s001.pdf]

Host strain: TG2

selection: KmAp | **selection: none**

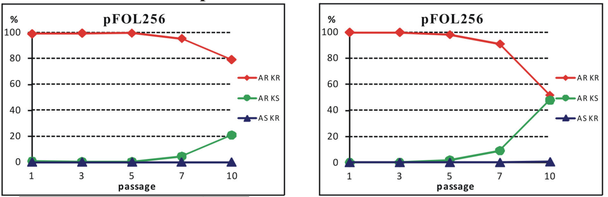

selection: KmAp

◆	99.21	99.46	99.66	95.44	79.08
●	0.79	0.54	0.34	4.56	20.92
▲	<0.23	<0.20	<0.08	<0.27	<0.42

selection: none

◆	100.00	100.00	98.20	91.06	51.78
●	<0.69	<0.21	1.80	8.94	47.74
▲	<0.69	<0.21	<0.23	<0.46	0.48

S1 Fig. Dynamics of IS*30*-mediated decomposition of the cointegrate-like plasmid pFOL256

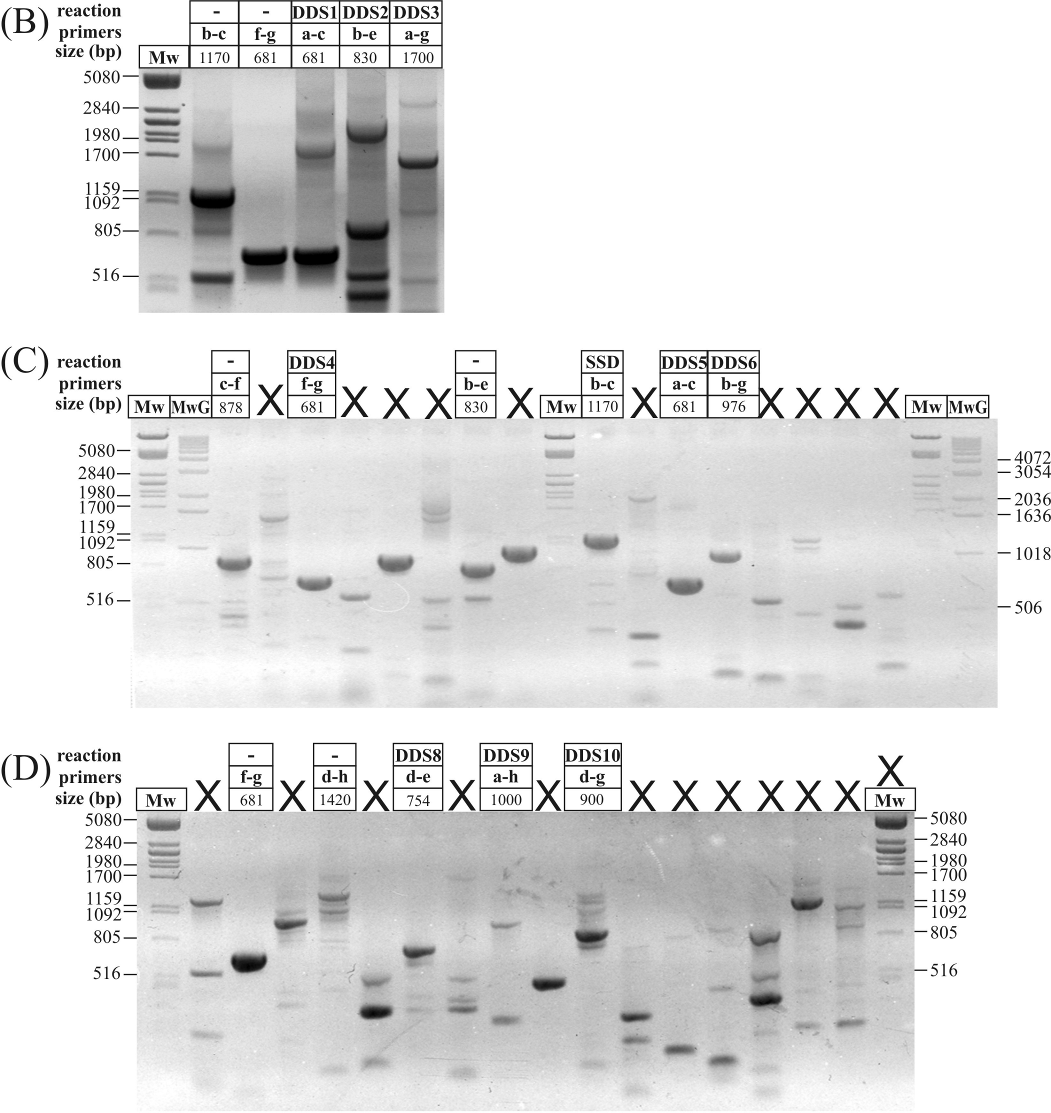

S2 Fig. Original gel images applied to compile panels B, C and D of **Fig 3**. Mw: λ DNA digested with *Pst*I; MwG: Gibco molecular weight ladder. Other symbols are as in Fig. 3.

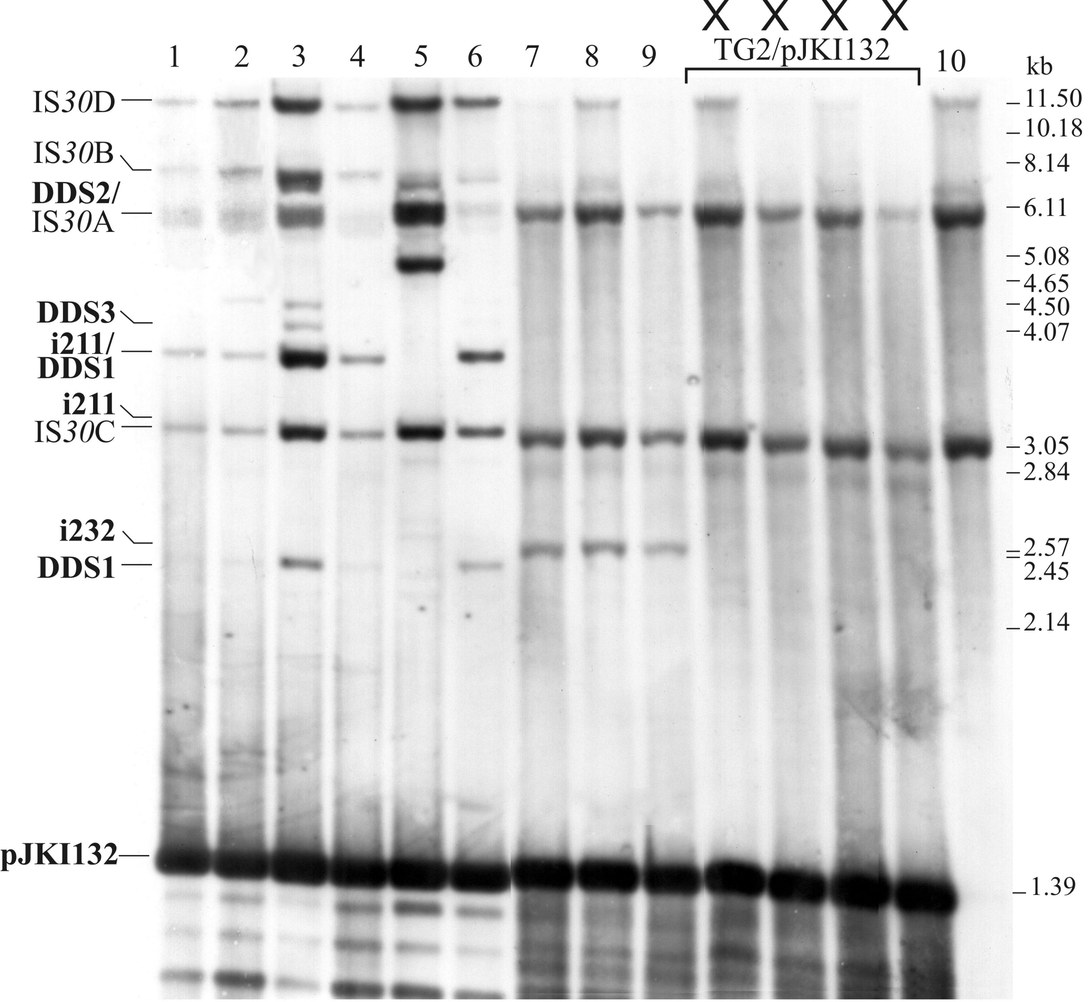

S3 Fig. Original image of Southern hybrisation applied to compile Fig. 4B. Lanes 1-10 and symbols are as in Fig. 4B.

S1_Table. The number of plasmid species identified in the course of passaging the cointegrates.

pAW1067 (TG2) — selection: KmAp

cointegrate/strain	passage	AR KR products		AR KS products		AS KR products		
pAW1067 (TG2)		pAW1067	del	pAW1124	del	pAW1038	pAW1039	sum
	1	20	0	1	0	0	0	21
	3	19	0	9	0	0	0	28
	5	19	1	12	0	0	1	33
	7	20	0	11	0	0	0	31
	10	20	0	8	1	1	0	30
								143

pAW1067 (TG2) — selection: none

passage	AR KR products		AR KS products		AS KR products		
	pAW1067	del	pAW1124	del	pAW1038	pAW1039	sum
1	20	0	0	0	0	0	20
3	20	0	11	0	0	0	31
5	18	1	20	0	0	1	40
7	20	0	16	0	0	0	36
10	20	0	20	0	1	0	41
							168

pAW1067 (JM109) — selection: KmAp

cointegrate/strain	passage	pAW1067	del	pAW1124	del	pAW1038	pAW1039	sum
pAW1067 (JM109)	1	20	0	3	0	0	0	23
	3	20	0	4	0	0	0	24
	5	20	0	0	0	0	0	20
	7	20	0	8	0	0	1	29
	10	20	0	12	1	2	1	36
								132

pAW1067 (JM109) — selection: none

passage	pAW1067	del	pAW1124	del	pAW1038	pAW1039	sum
1	20	0	4	0	0	0	24
3	20	0	9	0	0	0	29
5	19	0	9	0	0	1	29
7	20	0	12	0	0	0	32
10	20	0	20	0	1	0	41
							155

pAW1072 (TG2) — selection: KmAp

cointegrate/strain	passage	AR KR products			AR KS products		AS KR products		
pAW1072 (TG2)		pAW1072	pFOL257	del	pAW758	del	pAW1038	pAW1039	sum
	1	23	0	0	13	0	1	1	38
	3	21	1	0	12	0	1	3	38
	5	19	3	0	25	1	1	2	51
	7	17	2	1	21	1	1	2	45
	10	18	3	0	24	0	2	3	50
									222

pAW1072 (TG2) — selection: none

passage	AR KR products			AR KS products		AS KR products		
	pAW1072	pFOL257	del	pAW758	del	pAW1038	pAW1039	sum
1	20	0	0	20	0	0	0	40
3	19	1	0	20	0	0	0	40
5	20	3	0	20	0	0	0	43
7	18	5	0	19	0	0	0	42
10	18	4	0	20	0	0	0	42
								207

pAW1072 (JM109) — selection: KmAp

cointegrate/strain	passage	pAW1072	pFOL257	del	pAW758	del	pAW1038	pAW1039	sum
pAW1072 (JM109)	1	17	1	0	18	1	1	3	41
	3	19	0	1	17	0	2	2	41
	5	14	3	3	16	1	3	13	53
	7	21	2	1	21	1	9	13	68
	10	16	4	1	17	1	6	12	57
									260

pAW1072 (JM109) — selection: none

passage	pAW1072	pFOL257	del	pAW758	del	pAW1038	pAW1039	sum
1	18	2	0	18	0	1	1	40
3	19	1	1	21	0	2	1	45
5	18	2	0	19	1	3	11	54
7	22	2	0	21	0	10	9	64
10	14	4	3	19	1	7	8	56
								259

pFOL622 (TG2) — selection: KmAp

cointegrate/strain	passage	AR KR products			AR KS products			AS KR products		
pFOL622 (TG2)		pFOL622	pAW1067	del	pAW1124	del	inv	pAW1038	pAW1039	sum
	1	22	0	1	19	1	1	0	1	45
	3	21	1	1	19	0	4	1	0	47
	5	18	3	6	20	0	4	6	6	63
	7	14	5	5	19	1	5	5	5	59
	10	15	8	6	17	3	5	8	5	67
										281

pFOL622 (TG2) — selection: none

passage	AR KR products			AR KS products		AS KR products		
	pFOL622	pAW1067	del	pAW1124	del	pAW1038	pAW1039	sum
1	25	1	0	18	0	0	0	44
3	17	4	1	20	0	1	1	44
5	18	5	3	20	0	5	4	55
7	14	2	5	19	1	4	5	50
10	15	3	5	18	2	9	6	58
								251

pAW1118 TG2) — selection: KmAp

cointegrate/strain	passage	AR KR products				AR KS products		AS KR products		
pAW1118 TG2)		pAW1118	pFOL256	pFOL617	del	pAW1105	del	pAW1038	pAW1039	sum
	1	21	0	0	2	11	1	0	1	36
	3	20	0	2	3	19	0	1	1	46
	5	17	3	1	5	19	0	4	7	56
	7	15	3	4	5	19	1	5	7	59
	10	16	1	3	7	16	4	9	6	62
										259

pAW1118 TG2) — selection: none

passage	AR KR products			AR KS products			AS KR products		
	pAW1118	pFOL256	pFOL617	del	pAW1105	del	pAW1038	pAW1039	sum
1	19	0	0	1	17	1	1	0	39
3	21	1	1	2	18	0	0	2	45
5	16	2	2	4	19	0	3	4	50
7	16	3	3	3	20	1	6	8	60
10	17	1	2	6	17	5	8	5	61
									255

pAW256 TG2) — selection: KmAp

cointegrate/strain	passage	AR KR products				AR KS products			AS KR products	
pAW256 TG2)		pFOL256	pFOL255	del	inv	pAW1105	del	inv	pAW1038	sum
	1	19	0	0	0	18	0	0	0	37
	3	19	3	0	0	18	2	0	2	44
	5	17	2	1	0	18	0	1	5	44
	7	18	3	0	1	19	1	1	7	50
	10	16	2	0	0	19	1	0	8	46
										221

pAW256 TG2) — selection: none

passage	AR KR products			AR KS products		AS KR products	
	pFOL256	pFOL255	del	pAW1105	del	pAW1038	sum
1	25	3	0	1	0	0	29
3	16	4	0	4	1	2	27
5	17	3	1	5	4	3	33
7	17	3	0	15	9	5	49
10	18	4	1	15	8	4	50
							188

del - deletion derivatives
inv - inversion derivatives

S2 Table. Mutagen effect of an active chromosomal IR-IR junction on the metabolism of three sugars.

**Strain**	**IR-IR junction**	**Colonies tested**	**Mutants isolated**	**Frequency ($\times 10^{-4}$)**
		Maltose metabolism		
i211	+	41016	17	4.14
i231	+	64766	13	2.00
i1313	-	73429	0	<0.36
TG2	-	101495	0	<0.09
		Galactose metabolism		
i211	+	43529	17	3.91
i231	+	42407	19	4.48
i1313	-	62011	0	<0.16
TG2	-	98222	0	<0.10
		Xylose metabolism		
i211	+	35026	8	2.28
i231	+	35854	8	2.23
i1313	-	92787	0	<0.11
TG2	-	86106	0	<0.11
