# Supplementary material for: The dynamic network of IS30 transposition pathways
Source: PLoS One. 2022 Jul 28;17(7):e0271414. doi: 10.1371/journal.pone.0271414 (PMC9333248; doi:10.1371/journal.pone.0271414)
Supplement: S1 Raw images — (PDF) [file pone.0271414.s002.pdf]

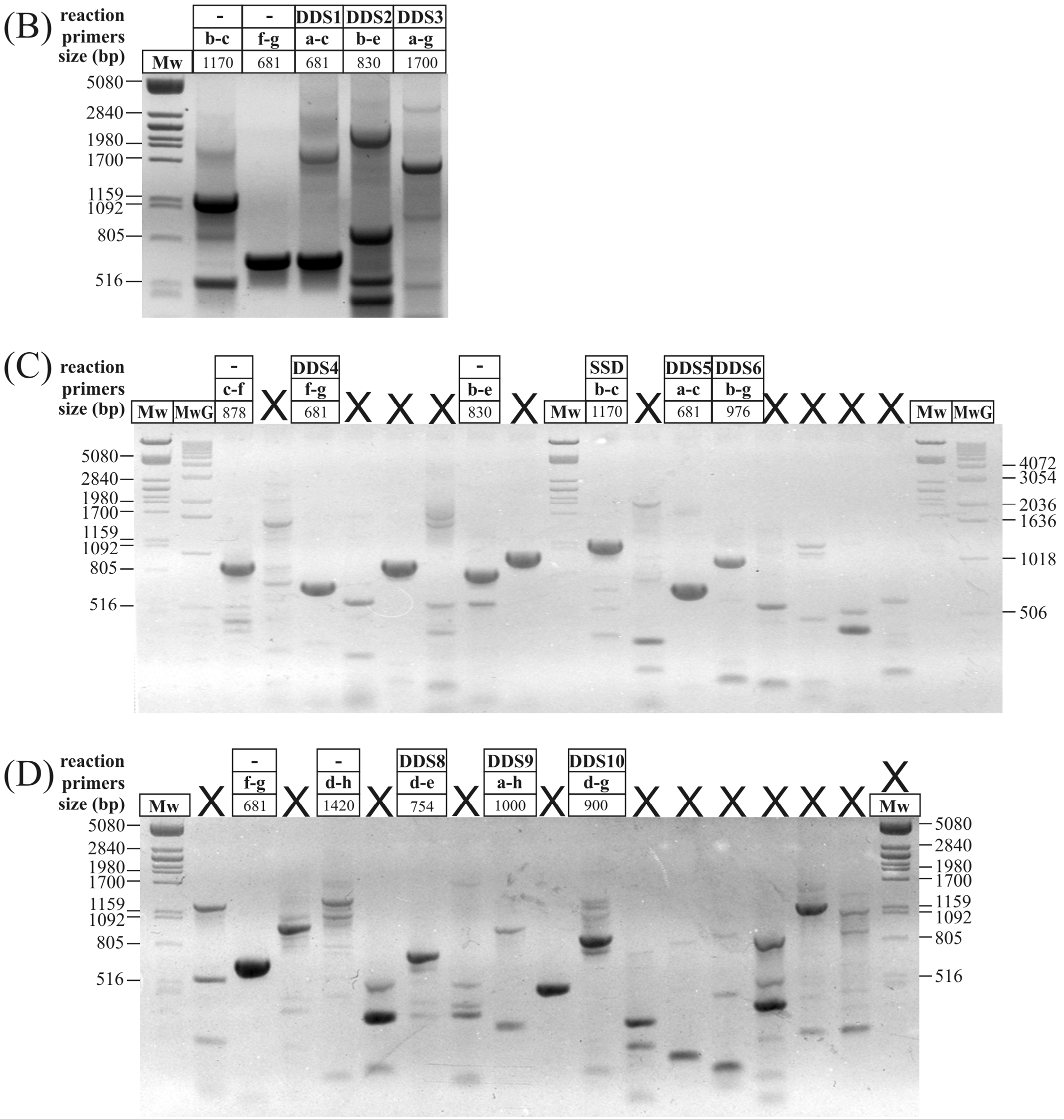

S2 Fig. Original gel images applied to compile panels B, C and D of **Fig 3**. Mw: λ DNA digested with *Pst*I; MwG: Gibco molecular weight ladder. Other symbols are as in Fig. 3.

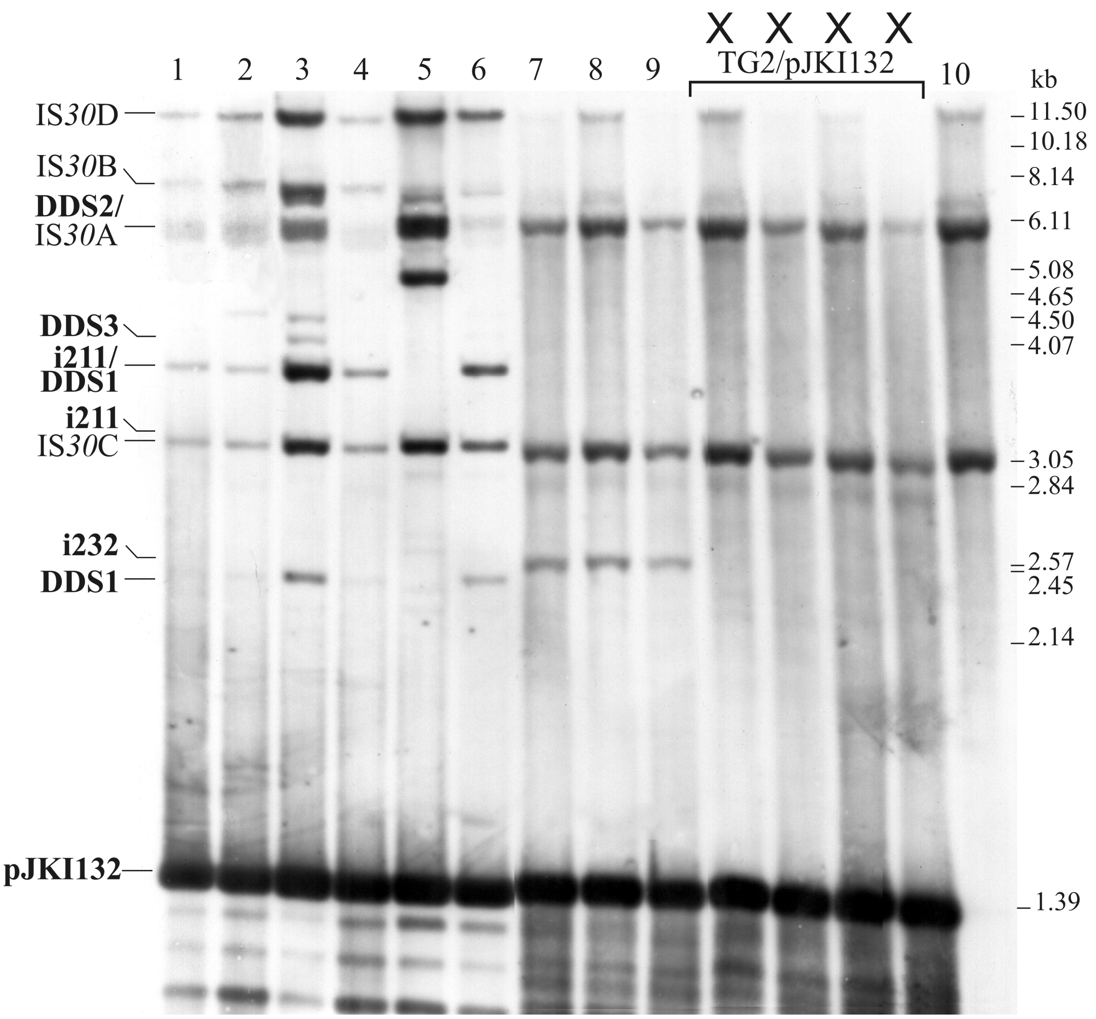

S3 Fig. Original image of Southern hybrisation applied to compile Fig. 4B. Lanes 1-10 and symbols are as in Fig. 4B.

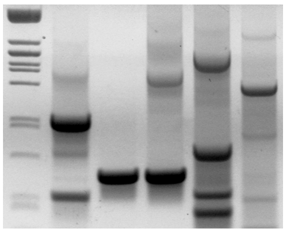

scanned negative photo of the EtBr-stained gel

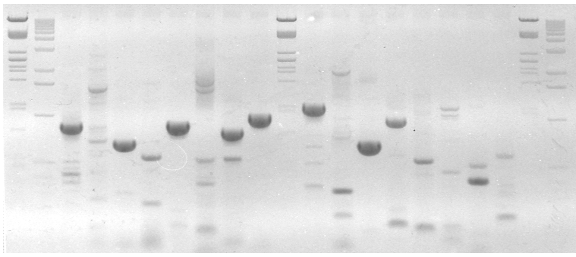

scanned negative photo of the EtBr-stained gel

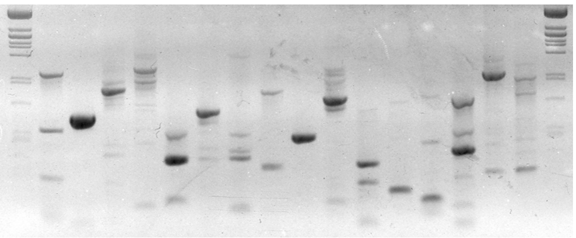

scanned negative photo of the EtBr-stained gel

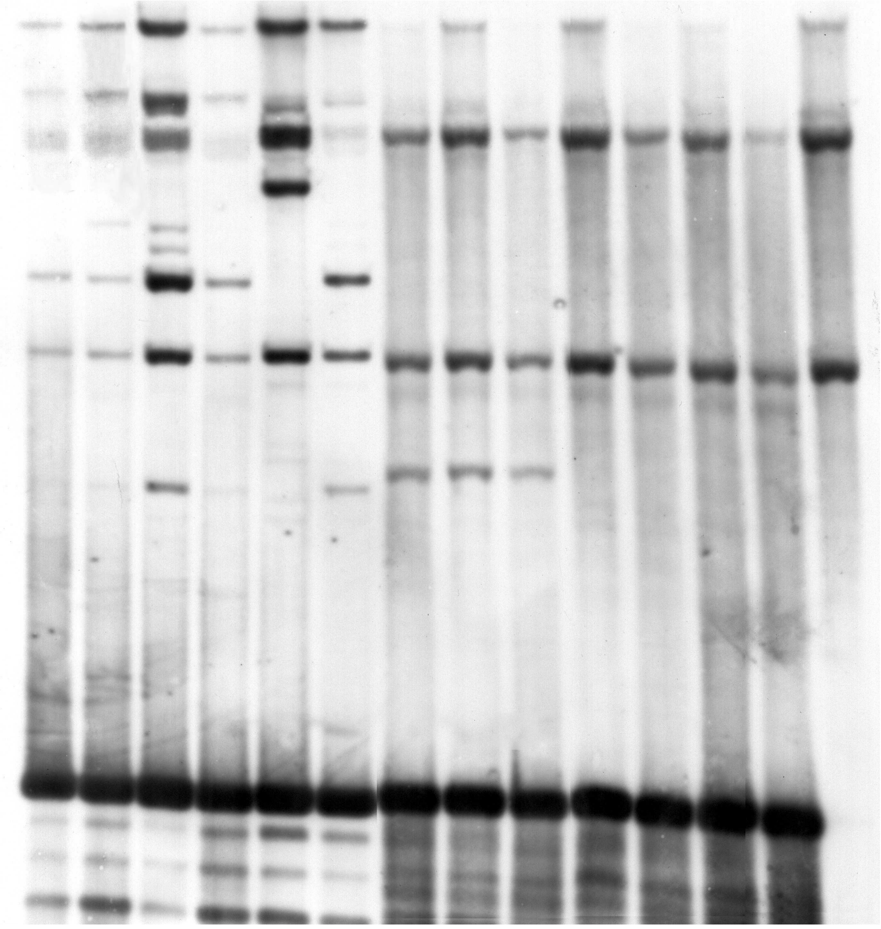

scanned image of the developed Southern blot filter
